# Cyclosporine A and Tacrolimus Induce Functional Impairment and Inflammatory Reactions in Endothelial Progenitor Cells

**DOI:** 10.3390/ijms22189696

**Published:** 2021-09-08

**Authors:** Nadia Meyer, Lars Brodowski, Constantin von Kaisenberg, Bianca Schröder-Heurich, Frauke von Versen-Höynck

**Affiliations:** 1 Gynecology Research Unit, Hannover Medical School, Carl-Neuberg-Strasse 1, D-30625 Hannover, Germany; Meyer.Nadia@mh-hannover.de (N.M.); Brodowski.Lars@mh-hannover.de (L.B.); Schroeder-Heurich.Bianca@mh-hannover.de (B.S.-H.); 2 Department of Obstetrics and Gynecology, Hannover Medical School, Carl-Neuberg-Strasse 1, D-30625 Hannover, Germany; vonKaisenberg.Constantin@mh-hannover.de

**Keywords:** endothelial colony-forming cells, endothelial progenitor cells, cyclosporine A, tacrolimus, endothelial dysfunction, cardiovascular risk, inflammation, transplantation

## Abstract

Immunosuppressants are a mandatory therapy for transplant patients to avoid rejection of the transplanted organ by the immune system. However, there are several known side effects, including alterations of the vasculature, which involve a higher occurrence of cardiovascular events. While the effects of the commonly applied immunosuppressive drugs cyclosporine A (CsA) and tacrolimus (Tac) on mature endothelial cells have been addressed in several studies, we focused our research on the unexplored effects of CsA and Tac on endothelial colony-forming cells (ECFCs), a subgroup of endothelial progenitor cells, which play an important role in vascular repair and angiogenesis. We hypothesized that CsA and Tac induce functional defects and activate an inflammatory cascade via NF-κB signaling in ECFCs. ECFCs were incubated with different doses (0.01 µM–10 µM) of CsA or Tac. ECFC function was determined using in vitro models. The expression of inflammatory cytokines and adhesion molecules was explored by quantitative real-time PCR and flow cytometry. NF-κB subunit modification was assessed by immunoblot and immunofluorescence. CsA and Tac significantly impaired ECFC function, including proliferation, migration, and tube formation. TNF-α, IL-6, VCAM, and ICAM mRNA expression, as well as PECAM and VCAM surface expression, were enhanced. Furthermore, CsA and Tac led to NF-κB p65 subunit phosphorylation and nuclear translocation. Pharmacological inhibition of NF-κB by parthenolide diminished CsA- and Tac-mediated proinflammatory effects. The data of functional impairment and activation of inflammatory signals provide new insight into mechanisms associated with CsA and Tac and cardiovascular risk in transplant patients.

## 1. Introduction

Immunosuppressant use is common in autoimmune, rheumatological, and chronic inflammatory bowel diseases, while their application is mandatory to prevent rejection after organ transplantation [1]. Frequently used immunosuppressants are cyclosporine A and tacrolimus. They prevent the activation of the phosphatase calcineurin. As a consequence, the nuclear factor of activated T cells cannot be dephosphorylated and translocated into the cell nucleus. As a result, T cell activation, transcription, and release of cytokines such as IL-2 are inhibited [2].

In addition to the intended mechanism, there are numerous side effects associated with their use. Endothelial dysfunction is one of the most recognized vascular impacts in patients after organ transplantation and is associated with nephrotoxicity, hypertension, hypercholesterinemia, and hyperglycemia [3,4].

While the impact of cyclosporine A and tacrolimus on mature endothelial cells has been addressed by different studies [5,6], it is barely known whether the functionality of endothelial progenitor cells (EPCs) is affected by them. Asahara et al. described and isolated EPCs for the first time in 1997 [7]. EPCs are impaired in several cardiovascular diseases [8,9,10] and considered a strong biomarker to determine endothelial dysfunction and cardiovascular risk [11,12,13,14]. Endothelial colony-forming cells (ECFCs), a subgroup of EPCs, are known for their high proliferative ability and play a crucial role in vascular repair and angiogenesis [15,16]. They contribute to endothelial integrity by incorporating into damaged vessel walls and further through paracrine effects [17,18]. At present, they are considered the most potent vascular reparative cell type among EPC subtypes [19].

A potent proangiogenic factor that regulates vascular growth and angiogenesis in EPCs is the vascular endothelial growth factor (VEGF). VEGF mediates its potentiating effect via AKT [20], a serine/threonine protein kinase that acts as a multifunctional intracellular regulator of cell growth, metabolism, and survival [21].

Inflammation is a hallmark of several diseases, e.g., cardiovascular disease [22] or preeclampsia [23], and is associated with higher cardiovascular risk in later life [24]. The activation of the protein complex nuclear factor kappa-light-chain-enhancer of activated B cells (NF-κB) is crucial for the regulation of the immune response and is involved in the moderation of inflammatory responses in the cardiovascular system [25,26]. NF-κB stimulates the transcription of proinflammatory cytokines, including tumor necrosis factor-alpha (TNF-α) and interleukin 6 (IL-6) [27,28]. Nuclear translocation of the NF-κB p65 subunit is a key event in NF-κB activation [29]. Furthermore, NF-κB coordinates the expression of adhesion molecules, including vascular cell adhesion protein (VCAM), intercellular adhesion molecule (ICAM), and platelet and endothelial cell adhesion molecule (PECAM), which are important regulators of leukocyte extravasation, vascular permeability, and inflammatory processes [28,30]. Whereas in T lymphocytes, cyclosporine A and tacrolimus reduce the activation of NF-κB [31], they have opposite effects in renal tubular cells [32] and in murine pancreatic islet endothelial cells [33]. Whether a similar NF-κB modification causing inflammation may happen in ECFCs exposed to cyclosporine A or tacrolimus has not been addressed yet.

In light of the increased cardiovascular risk of transplant patients and ECFCs’ key role in vascular repair and homeostasis, in this study, we investigate the effects of cyclosporine A and tacrolimus on the functional properties of ECFCs and on inflammatory cascades.

## 2. Results

### 2.1. High-Dose Cyclosporine A and Tacrolimus Inhibit ECFC Proliferation

**Figure 1:**
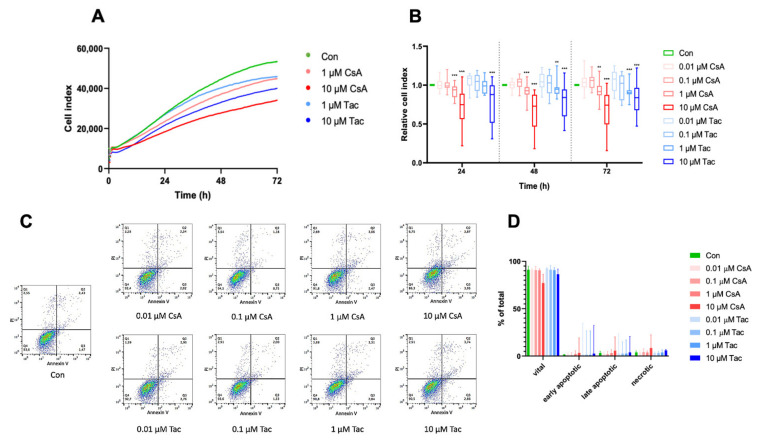
High-dose cyclosporine A (CsA) and tacrolimus (Tac) impaired ECFC proliferation. (**A**) Overlay of growth curves of ECFCs treated with CsA or Tac (1 µM or 10 µM). (**B**) ECFC proliferation was not affected after treatment with 0.01 or 0.1 µM CsA or Tac, but it was significantly decreased after treatment with 1 µM or 10 µM CsA or Tac after 24 h, 48 h, and 72 h. *n* = 13–27; control group set as 1. (**C**) Representative measurement of apoptosis and necrosis in ECFCs after 48 h treatment with CsA or Tac at 0.01 µM, 0.1 µM, 1 µM, or 10 µM. Viable cells are located in the lower left field (Annexin V neg./PI neg). (**D**) There was no significant increase of apoptotic or necrotic cells. *n* = 5. Con—control; CsA—cyclosporine A; Tac—tacrolimus. ** *p* < 0.01, *** *p* < 0.001.

Regarding one of their main characteristics, we analyzed ECFCs’ proliferation in the presence or absence of different doses of cyclosporine A or tacrolimus. The cell index was continuously monitored with the xCelligence technology. Treatment with 0.01 µM or 0.1 µM cyclosporine A or tacrolimus did not affect ECFCs’ proliferation, whereas treatment with 1 µM or 10 µM significantly decreased ECFCs’ proliferation after 24 h, 48 h, and 72 h, respectively (Figure 1A,B, Supplemental Tables S1–S3). Less pronounced but comparable effects were also observed at shorter exposures of 14 h and 18 h, time points which were used in the migration and angiogenesis assays (Supplemental Figure S1, Supplemental Tables S4 and S5).

To explore whether apoptosis is involved in functional impairments observed after treatment with different doses of cyclosporine A or tacrolimus, we determined the expression level of the apoptosis marker annexin V using flow cytometry. After 48 h of incubation, there was no significant difference in apoptosis or necrosis in ECFCs treated with cyclosporine A or tacrolimus compared to vehicle control (Figure 1C,D, Supplemental Tables S6–S8).

### 2.2. Cyclosporine A and Tacrolimus Impair ECFC Migration

**Figure 2:**
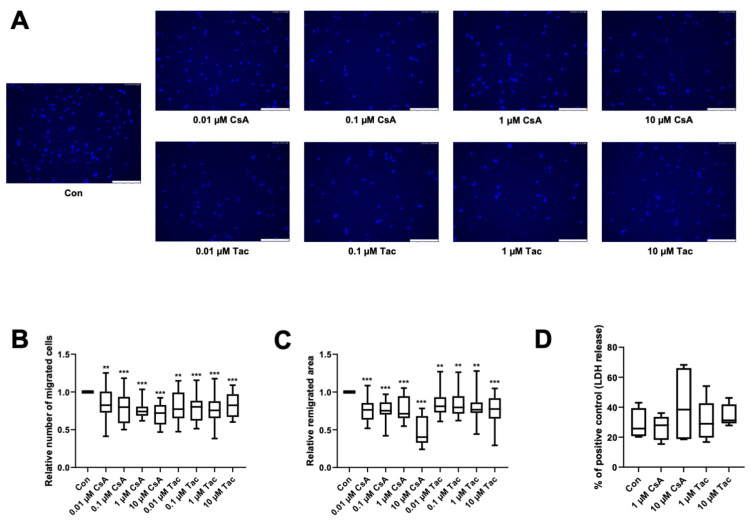
Cyclosporine A (CsA) and tacrolimus (Tac) impaired ECFC migration. (**A**) Representative images of DAPI-stained migrated ECFCs treated with CsA or Tac (0.01–10 µM) in a chemotaxis assay, scale bar 150 µm. (**B**) ECFCs showed a significantly lower directional migration in presence of CsA or Tac after 4 h. Numbers of DAPI-stained migrated cells on the bottom side of the membranes were counted in each picture, control group set as 1. *n* = 20. (**C**) CsA or Tac treatment (0.01–10 µM) of ECFCs diminished wound closure assessed as remigrated area after 18 h compared to control, control group set as 1. Representative images can be found in Supplemental Figure S2. *n* = 15–16. (**D**) CsA or Tac (1 µM or 10 µM) did not lead to significantly higher LDH release after 18 h, positive control set as 100%, *n* = 9. Con—control; CsA—cyclosporine A; Tac—tacrolimus. ** *p* < 0.01, *** *p* < 0.001.

The directional migration of single cells is driven by a chemotactic gradient and was tested using a modified Boyden chamber assay. The chemotaxis ability of ECFCs was significantly lower when treated with cyclosporine A (relative number of migrated cells: 0.01 µM: 0.83, *p* = 0.02; 0.1 µM: 0.80, *p* < 0.001; 1 µM: 0.74, *p* < 0.001; 10 µM: 0.72, *p* < 0.001) or tacrolimus (relative number of migrated cells: 0.01 µM: 0.77, *p* = 0.02; 0.1 µM: 0.80, *p* < 0.001; 1 µM: 0.76, *p* < 0.001; 10 µM: 0.82, *p* < 0.001) (Figure 2A,B, Supplemental Table S9).

As cyclosporine A and tacrolimus have been shown to decrease the directional migration of ECFCs towards a chemoattractant, we further addressed if they also influence ECFCs’ remigration of a scratch wound in a former monolayer. Wound closure was significantly lower in the presence of cyclosporine A (relative remigrated area: 0.01 µM: 0.76; 0.1 µM: 0.75; 1 µM: 0.71; 10 µM: 0.40, all *p* < 0.001) and tacrolimus (relative remigrated area: 0.01 µM: 0.81, *p* = 0.003; 0.1 µM: 0.80, *p* = 0.002; 1 µM: 0.77, *p* = 0.002; 10 µM: 0.78, *p* < 0.001) (Figure 2C, Supplemental Figure S2, Supplemental Table S10).

To exclude cytotoxicity as the cause of the detected functional impairment, we performed an LDH assay with the supernatants of the scratch wound healing assay. There was no significant increase of LDH release after 18 h of incubation with 1 or 10 µM cyclosporine A or tacrolimus compared to vehicle control (Con: 25.78%; 1 µM cyclosporine A: 27.96%, *p* = 0.73; 10 µM cyclosporine A: 38.43%, *p* = 0.61; 1 µM tacrolimus: 28.94%, *p* = 0.73; 10 µM tacrolimus: 31.22%, *p* = 0.08). All values were related to the positive control (Figure 2D, Supplemental Table S11).

### 2.3. Cyclosporine A and Tacrolimus Decrease ECFC Angiogenic Capacity and AKT Phosphorylation

**Figure 3:**
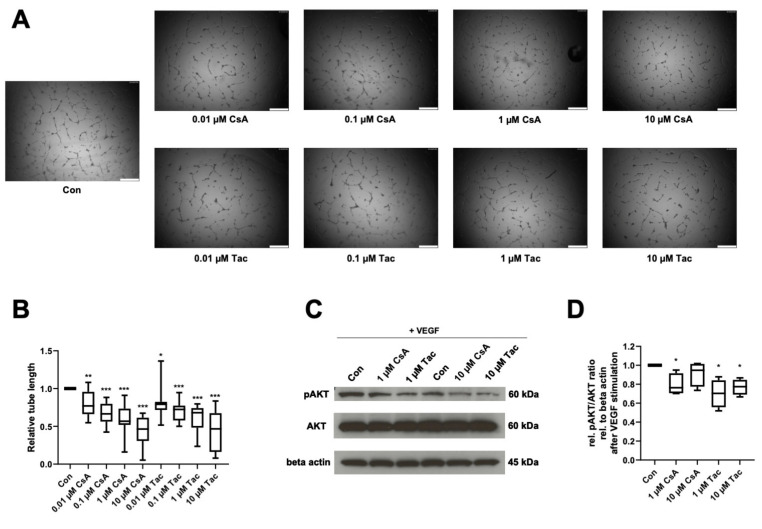
Cyclosporine A (CsA) and tacrolimus (Tac) impaired ECFC angiogenesis in presence of VEGF and reduced AKT phosphorylation. (**A**) Representative images from ECFCs treated with vehicle control only, 0.01 µM CsA, 0.1 µM CsA, 1 µM CsA, 10 µM CsA, 0.01 µM Tac, 0.1 µM Tac, 1 µM Tac, 10 µM Tac for 14 h, scale bar 500 µm. (**B**) CsA and Tac treatment of ECFCs significantly decreased tube length after 14 h of incubation. Control group set as 1. *n* = 12. (**C**) Representative immunoblot of AKT phosphorylation in the presence of CsA or Tac (1 or 10 µM) after 10 min of stimulation with VEGF. (**D**) CsA and Tac (1 or 10 µM) prevented the VEGF-induced AKT phosphorylation in ECFCs. Control group set as 1. *n* = 4. Con—control; CsA—cyclosporine A; Tac—tacrolimus. *, *p* < 0.05, ** *p* < 0.01, *** *p* < 0.001.

Reflecting ECFCs’ ability of de novo vessel formation in vivo, we performed an in vitro angiogenesis assay to determine cyclosporine A and tacrolimus effects in presence of VEGF on ECFCs’ ability to form capillary-like structures in Matrigel. After treatment with cyclosporine A or tacrolimus, ECFCs showed significantly shorter tube length than controls (cyclosporine A: 0.01 µM: 0.77, *p* = 0.005; 0.1 µM: 0.67, *p* < 0.001; 1 µM: 0.57, *p* < 0.001; 10 µM: 0.47, *p* < 0.001; tacrolimus: 0.01 µM: 0.79, *p* = 0.02; 0.1 µM: 0.72, *p* < 0.001; 1 µM: 0.68, *p* < 0.001; 10 µM: 0.47, *p* < 0.001) (Figure 3A,B, Supplemental Table S12).

As AKT belongs to the main targets of VEGF and the AKT-cascade is considered an important signaling pathway in endothelial cells, we addressed possible effects of cyclosporine A and tacrolimus on AKT phosphorylation in ECFCs. Cyclosporine A (1 µM) and tacrolimus (1 or 10 µM) reduced AKT phosphorylation after VEGF stimulation compared to control (cyclosporine A: 1 µM: 0.76, *p* = 0.04; 10 µM: 0.95, *p* = 0.27; tacrolimus: 1 µM: 0.70, *p* = 0.03; 10 µM. 0.78, *p* = 0.01) (Figure 3C,D, Supplemental Table S13).

### 2.4. Cyclosporine A and Tacrolimus Affect Expression of Inflammatory Cytokines and Adhesion Molecules

**Figure 4:**
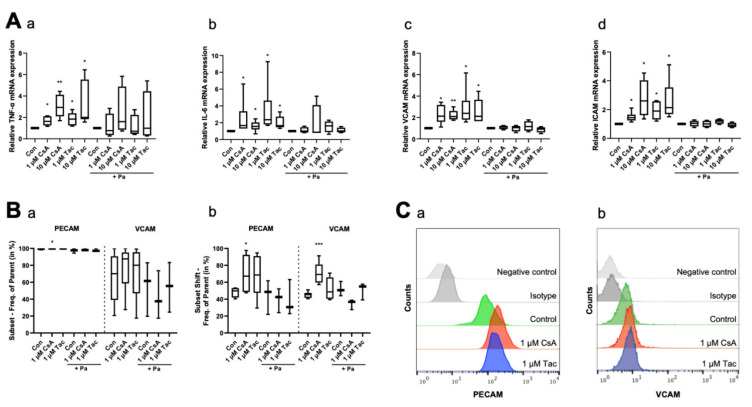
Cyclosporine A (CsA) and tacrolimus (Tac) increased mRNA and cell surface expression of inflammatory cytokines and adhesion molecules in ECFCs. (**A**) CsA and Tac (1 or 10 µM) significantly induced mRNA expression of the inflammatory cytokines TNF-α (a) and IL-6 (b) and of the adhesion molecules VCAM (c) and ICAM (d). Parthenolide (Pa), an NF-κB inhibitor, reduced the CsA- and Tac-induced effects. All runs were performed in triplicates. Control group set as 1, *n* = 4–6. In flow cytometry (**B**), treatment with 1 µM CsA significantly increased PECAM and VCAM cell surface expression (a) and shift (b), treatment with 1 µM Tac only showed a slight increase. Pa moderated the CsA- and Tac-induced effects, *n* = 3–6. (**C**) Representative measurement of PECAM (a) and VCAM (b) surface expression after treatment with 1 µM CsA or 1 µM Tac. Con—control; CsA—cyclosporine A; Tac—tacrolimus; Pa—parthenolide. * *p* < 0.05, ** *p* < 0.01, *** *p* < 0.001.

To explore the inflammatory reactions in ECFCs, we determined the mRNA levels of the inflammatory cytokines TNF-α and IL-6, as well as VCAM and ICAM mRNA levels, as markers of adhesion molecules under inflammatory conditions, by using qRT-PCR after 6 h of treatment with cyclosporine A or tacrolimus. ECFCs treated with cyclosporine A or tacrolimus (1 or 10 µM) showed a higher expression level of TNF-α (cyclosporine A: 1 µM: 1.63, *p* = 0.03; 10 µM: 2.94, *p* = 0.01; tacrolimus: 1 µM: 1.85, *p* = 0.03; 10 µM: 2.00, *p* = 0.03) and IL-6 (cyclosporine A: 1 µM: 1.67, *p* = 0.03; 10 µM: 1.56, *p* = 0.02; tacrolimus: 1 µM: 2.37, *p* = 0.03; 10 µM: 1.65, *p* = 0.03) compared to controls. The addition of the NF-κB inhibitor parthenolide attenuated the effect (Figure 4A, Supplemental Tables S14 and S15). Furthermore, treatment with cyclosporine A or tacrolimus led to a higher mRNA expression level of the adhesion molecules VCAM (cyclosporine A: 1 µM: 2.13, *p* = 0.02; 10 µM: 2.09, *p* = 0.01; tacrolimus: 1 µM: 2.39, *p* = 0.03; 10 µM: 2.11, *p* = 0.04) and ICAM (cyclosporine A: 1 µM: 1.44, *p* = 0.02; 10 µM: 2.60, *p* = 0.02; tacrolimus: 1 µM: 1.90, *p* = 0.01; 10 µM: 2.14, *p* = 0.03). The addition of parthenolide again attenuated the effect (Figure 4A, Supplemental Tables S16 and S17). Regarding the surface expression of adhesion molecules, we observed a higher PECAM surface expression after a 12 h treatment with 1 µM cyclosporine A (subset: 99.80%, *p* = 0.04; subset shift: 67.20%, *p* = 0.047) via flow cytometry compared to vehicle control (subset: 99.65%, subset shift: 49.75%). Treatment with 1 µM tacrolimus showed a similar trend but no significant effect (subset: 99.85%, *p* = 0.12; subset shift: 68.65%, *p* = 0.10). We further detected a marked but not significant increase of VCAM surface expression after treatment with 1 µM cyclosporine A (subset: 87.60%, *p* = 0.48) or tacrolimus (subset: 80.10%, *p* = 0.75) compared to vehicle control (subset: 70.20%). The cyclosporine A effect was especially pronounced in the subset shift (69.30%, *p* < 0.001), whereas tacrolimus did not affect the subset shift at all (48.65%, *p* = 0.25) compared to vehicle control (45.35%). Parthenolide diminished these effects. Treatment with parthenolide alone caused no significant effect (Figure 4B,C, Supplemental Tables S18–S21).

### 2.5. Cyclosporine A and Tacrolimus Induce Phosphorylation and Nuclear Translocation of NF-κB p65 Subunit

**Figure 5:**
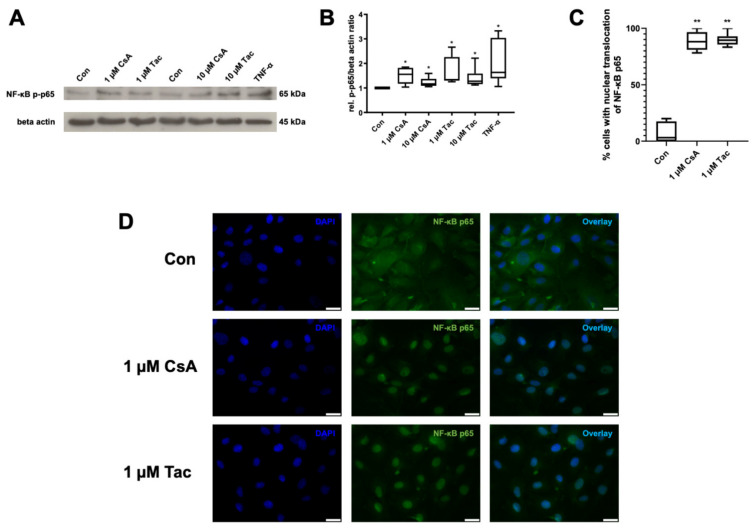
Cyclosporine A (CsA) and tacrolimus (Tac) induced phosphorylation and nuclear translocation of NF-κB p65 subunit in ECFCs. (**A**) Representative immunoblot of NF-κB p65 phosphorylation after 30 min treatment with CsA or Tac (1 or 10 µM). TNF-α was used as positive control. (**B**) CsA and Tac significantly increased NF-κB p65 subunit phosphorylation in ECFCs, *n* = 6, control group set as 1. (**C**) Quantification of ECFCs showing nuclear translocation of NF-κB p65 subunit after treatment with 1 µM CsA or 1 µM Tac for 30 min compared to vehicle control. Cells were counted in 6 randomly taken images per treatment. (**D**) Representative immunofluorescence images showing NF-κB p65 (green) nuclear translocation of ECFCs treated with CsA or Tac (1 µM) for 30 min, nuclei were counterstained with DAPI (blue), scale bar 25 µm, *n* = 3. Con—control; CsA—cyclosporine A; Tac—tacrolimus. * *p* < 0.05, ** *p* < 0.01, control group set as 1.

NF-κB is of high importance for the regulation of immune responses. As the phosphorylation and nuclear translocation of its p65 subunit mark key events in its activation, we targeted possible effects of cyclosporine A or tacrolimus on NF-κB as a mediator of inflammatory reactions in ECFCs. Cyclosporine A and tacrolimus (1 or 10 µM) significantly increased NF-κB p65 phosphorylation compared to control (cyclosporine A: 1 µM: 1.55; 10 µM 1.17; tacrolimus: 1 µM: 1.39; 10 µM: 1.28, *p* = 0.03). TNF-α as a known inducer of NF-κB activity was used as positive control and displayed a 1.63-fold higher NF-κB p65 phosphorylation than the control (*p* = 0.03) (Figure 5A,B, Supplemental Table S22).

Furthermore, we detected a nuclear translocation of the NF-κB p65 subunit via immunofluorescence after incubation with cyclosporine A or tacrolimus (1 µM), whereas vehicle-treated ECFCs showed a cytoplasmatic distribution of NF-κB p65 subunit (Figure 5C,D, Supplemental Table S23).

## 3. Discussion

In this study, we provide evidence for functional impairment of ECFCs induced by cyclosporine A and tacrolimus. In addition to migration, proliferation, and angiogenesis defects, these immunosuppressants led to a proinflammatory reaction of ECFCs as observed by an increase of gene expression of the inflammatory cytokines TNF-α and IL-6, as well as of the adhesion molecules VCAM and ICAM. Furthermore, we demonstrated modulation of VCAM and PECAM on the ECFC surface. We finally found that this inflammatory reaction was based on a drug-induced modification of NF-κB p65.

Endothelial dysfunction is a preceding factor in the development of cardiovascular disease, with vascular damage and inflammation leading to atherogenesis [34,35]. Endothelial damage is mediated by a number of biological stimuli, such as inflammatory mediators and hypoxia [36]. In transplant patients, endothelial dysfunction is one of the most recognized vascular affections. Cyclosporine A and tacrolimus, essential to avoid graft rejection, are considered the main contributors [3,4].

EPCs were identified as endogenous endothelial repair mechanisms mitigating risk factor-induced effects and replacing dysfunctional endothelium [9]. They are impaired in number and functional capacity in heart failure [8,9,37] and hypertension [10], and they inversely correlate with risk factors for coronary artery disease [14]. Therefore, growing evidence suggests that EPCs could be a link between a defective homeostatic or endogenous repair mechanism and vascular dysfunction [38] and are now considered as one of the strongest biomarkers to evaluate endothelial dysfunction [11,12].

In our study, cyclosporine A and tacrolimus impaired key cellular functions of ECFCs, a proliferative subgroup of EPCs, which is in line with findings reported for other cell types. This includes a decreased proliferation of cardiomyocytes and endothelial cells derived from human embryonic stem cells after cyclosporine A or tacrolimus treatment [39]. Furthermore, cyclosporine A inhibited proliferation in human umbilical endothelial, human microvascular, and human renal tubular epithelial cells [40,41]. In renal tubular epithelial cells, cyclosporine A led to cell cycle arrest and inhibited DNA synthesis [42]. Due to their involvement in vascular repair, ECFCs have been identified as a promising model for cell-based therapy, e.g., in ischemia, and have been addressed as a therapeutic target in several studies [12,43,44,45,46]. In this context, it has already been demonstrated that cyclosporine A significantly lowers the therapeutic potency of ECFCs. While researchers originally hypothesized that immunosuppression would favor ECFCs’ potential of incorporation into damaged vessel walls, it turned out that cyclosporine A caused a significant decrease in reperfusion and poorer limb survival in mice [47]. While cyclosporine A and tacrolimus exert their protective, immunosuppressive effects via calcineurin inhibition, there is some evidence that endothelial dysfunction is independent of it [48].

Cardiovascular ischemia usually results in an increase in VEGF as a physiological response [49,50]. VEGF is known to display various effects on endothelial cells and is considered the master regulator of vascular growth [21,51]. Cyclosporine A and tacrolimus have been shown to block these VEGF-induced effects. For example, cyclosporine A and tacrolimus inhibited VEGF-induced migration and angiogenesis in different endothelial cells [49,52]. This is consistent with our findings that cyclosporine A and tacrolimus hindered VEGF-mediated tube formation. We further demonstrated that cyclosporine A and tacrolimus abrogated VEGF-induced phosphorylation of AKT, a multifunctional intracellular regulator of cell growth, metabolism and survival [21]. This is in line with a study that reported tacrolimus-induced inhibition of AKT phosphorylation of human umbilical vein endothelial cells prior to VEGF stimulation [53]. Furthermore, tacrolimus-mediated AKT inhibition caused tube breakdown in endothelial cells, suggesting that tacrolimus induces endothelial dysfunction through attenuation of AKT [48].

Inflammatory activation is a critical component of endothelial dysfunction and atherogenesis [54,55]. Apart from their angiogenic properties, ECFCs have also been shown to provide anti-inflammatory effects. Collett et al. reported that ECFC-conditioned medium significantly reduced the expression of adhesion molecules and decreased the number of differentiated lymphocytes typically recruited into the kidney following renal ischemia [16]. Furthermore, limb wounds treated with ECFCs showed higher blood vessel quantity in the wound periphery and lower density of neutrophils and macrophages [56]. However, little is known about whether and under what circumstances ECFCs can also harm or contribute to inflammation.

In our study, cyclosporine A and tacrolimus induced the transcription of the inflammatory cytokines TNF-α and IL-6 in ECFCs. This is consistent with the findings of Rodrigues Diez et al., who reported an increase of both cytokines in murine endothelial cells after treatment with these immunosuppressants [33]. Inflammatory cytokines secreted in response to cyclosporine A and tacrolimus may therefore result in an autocrine or paracrine loop leading to further endothelial activation, cytokine production, and deterioration of vascular function [33,57]. They promote endothelial dysfunction, arterial wall remodeling, arterial stiffening, atherosclerosis, and hypertension [58,59,60]. The proinflammatory cytokine TNF-α is highly augmented in several cardiovascular and inflammatory diseases and is considered a risk factor for developing these clinical conditions [61]. Regarding ECFCs, TNF-α was shown to inhibit proliferation, migration, and tube formation and further led to barrier breakdown [62,63]. Interestingly, ECFCs isolated from patients with systemic lupus erythematodes, an autoimmune inflammatory disease associated with higher cardiovascular risk, showed an elevated expression of IL-6 and were subsequently impaired in their basic physiological function, including proliferation, adhesion, migration, and tube formation [64].

In addition to the stimulation of inflammatory cytokine release, an increased expression of adhesion molecules can be interpreted as an additional sign of inflammatory activation in our study. An enhanced expression of adhesion molecules such as VCAM and ICAM is regarded as a manifestation of endothelial dysfunction accompanied by leukocyte infiltration into the vessel wall and is considered a consequence of chronic exposure to inflammatory cytokines [65,66,67]. We have already shown the ability of ECFCs to express adhesion molecules in previous studies [63,68]. ECFCs secrete proinflammatory mediators and can also upregulate proinflammatory adhesion molecules upon stimulation similar to mature endothelial cells [69], and activated ECFCs increase chemotactic gene expression, synthesize chemotaxis mediators, and favor leukocyte recruitment [70]. The influence of cyclosporine A and tacrolimus on adhesion molecule expression has been reported controversially. Lehle et al. stated that cyclosporine A and tacrolimus do not affect adhesion molecule expression of human macro- and microvascular endothelial cells and are therefore unlikely to contribute to endothelial cell activation in transplant-associated vasculopathy [71], and Sasakawa et al. described tacrolimus-mediated inhibition of VCAM and ICAM expression on human microvascular endothelial cells [72]. In contrast, Badiwala et al. found a cyclosporine A- and tacrolimus-induced increase in ICAM expression in human coronary endothelial cells [73], and Kidokoro et al. described that expression of ICAM mRNA was enhanced in glomeruli after tacrolimus treatment and resulted in macrophage infiltration into the glomeruli. Consequently, they hypothesized that cyclosporine A- and tacrolimus-induced inflammatory reactions could play a role in vascular injury and in complications associated with their long-term use [74]. Rodrigues Diez et al. found a significant cyclosporine A- and tacrolimus-induced increase of VCAM and ICAM mRNA expression and further demonstrated a higher ICAM-release of murine endothelial cells [33]. Apart from gene transcription and surface expression, a possible cyclosporine A- and tacrolimus-induced release of inflammatory cytokines through ECFCs should be targeted in further studies. With our current results, we provide another piece of the puzzle to explain the development of cardiovascular complications in cyclosporine A and tacrolimus therapy and support the hypothesis that the immune activity of EPCs might play a pathophysiologic role in the evolution of cardiovascular disease [75].

Based on the observed nuclear translocation and phosphorylation of the NF-κB p65 subunit, we further suggest that inflammatory reactions in ECFCs may be mediated via NF-κB. This is consistent with the available literature indicating that endothelial dysfunction generated by inflammatory cytokines is dependent on the NF-κB pathway [76]. NF-κB activation can induce the transcription of a large number of inflammatory genes in vascular cells and other cell types, including cytokines, chemokines, and adhesion molecules [25]. NF-κB expression correlates with upregulation of chemokines in human cardiovascular disease [25,77,78]. Therefore, activation of NF-κB is considered to contribute to vascular injury, hypertension, and systemic inflammation and further promotes atherosclerosis [79,80,81,82]. Badiwala et al. reported that cyclosporine A upregulated NF-κB p65 activity in human coronary endothelial cells [73]. Especially the phosphorylation at Serine 536, which we have shown to be inducible by cyclosporine A and tacrolimus as well, is suggested to be critical for increased NF-κB and subsequent NF-κB-dependent gene activation [83] and associated with a preserved inflammatory gene expression [33]. Nevertheless, whether treatment with cyclosporine A or tacrolimus leads to complete activation of NF-κB was not revealed in our study and would be of great interest for future research. The NF-κB inhibitor parthenolide attenuated cyclosporine A- and tacrolimus-induced inflammatory effects in ECFCs. It has been shown that pharmacological inhibition of NF-κB activation ameliorates hypertension, cardiovascular injury, and atherogenesis [81,84,85]. NF-κB suppression may therefore represent a therapeutic approach facing cardiovascular complications in transplant patients. Another promising substance group consists of statins. Statins are a class of lipid-lowering drugs that reduce morbidity and mortality in patients at high cardiovascular risk [86,87] and provide several beneficial pleiotropic effects, including anti-atherothrombotic properties and immune modulation [88,89]. Statin treatment prevented TNF-α-induced NF-κB activation and the nuclear translocation of the NF-κB p65 subunit in human endothelial cells [90] and in mice [91]. We recently identified the HMG-CoA reductase inhibitor pravastatin as a promising potentiator of ECFC function [92]. Further investigations may address to what extent statins are able to block cyclosporine A- and tacrolimus-induced functional impairment and inflammatory reactions in ECFCs.

As our study is based on in vitro data, the results cannot be transferred directly to the in vivo situation, and should be confirmed under in vivo conditions. The first approach in this context could be the investigation of ECFCs derived from individuals taking cyclosporine A or tacrolimus, which is currently explored by our group.

In conclusion, we demonstrate the induction of functional defects of ECFCs as well as the stimulation of inflammatory signals by cyclosporine A and tacrolimus. Since ECFCs are important players in vascular repair, these effects may be an explanatory approach for poor vascular health in transplant patients. The interaction of inflammation and functional capacities requires further investigation. Anti-inflammatory therapies may provide an option to improve vascular health in those affected. Our results consequently contribute to mechanistic explanations linking hypertension, vascular disease, and metabolic alterations observed in patients under long-term cyclosporine A or tacrolimus treatment.

## 4. Materials and Methods

### 4.1. ECFC Isolation, Culture, and Characterization

The study was approved by the Institutional Review Board of Hannover Medical School (approval no. 1443-2012). Written informed consent was obtained from each participant. Umbilical cord blood from 9 healthy, uncomplicated pregnancies was collected immediately after delivery. All pregnancies were normotensive and without proteinuria, ending with the delivery of a healthy singleton. None of the control patients referred a clinical history of diabetes or a hypertensive, vascular, or renal disease, smoking or the use of illicit drugs.

ECFCs were isolated as previously described by our group [93]. Density gradient centrifugation was performed to isolate the cord blood mononuclear cell (CBMC) fraction. Up to 1 × 10^7^ cells per well were plated in endothelial growth medium 2 (EGM-2) consisting of endothelial basal medium (EBM-2; Lonza, Basel, Switzerland) supplemented with supplier provided supplements, 10% fetal bovine serum (FBS; Harvard Bioscience, Holliston, MA, USA) and 1% penicillin/streptomycin (P/S; Bio&Sell, Feucht, Nürnberg, Germany) on collagen-coated 6-well plates (BioCoat; Corning, NY, USA) and incubated at 37 °C, 5% CO_2_ supply. First ECFC colonies were noted as circumscribed monolayers of rapidly proliferating cells with cobblestone morphology and characterized by flow cytometry with the appropriate antibodies (CD31 (130-117-390; BD Biosciences, San Jose, CA, USA), CD45 (555483; BD Biosciences), and CD133 (130-090-826; Miltenyi Biotec, Bergisch Gladbach, Germany)) and the corresponding isotype controls (BD Biosciences, Miltenyi Biotec). ECFCs were used for experiments in cell culture passages 4–6.

### 4.2. Cell Impedance Assay

The cell index, a parameter reflecting cell size, adhesion, and proliferation, was calculated with an xCelligence Real-Time Cell Analyzer (Roche, Basel, Switzerland), which allows continuous cell monitoring in real time. The electrical impedance caused by adherent cells is converted into cell indices by the xCelligence software (v.1.2.1). Then, 0.25 × 10^4^ cells of 5 ECFC lines were seeded in quadruplicates in EGM-2 with 8% FBS and 1% P/S onto a gold-coated E-Plate View 96-well plate (Roche) and then placed into the Real-Time Cell Analyzer SP station, positioned in a 37 °C incubator with 5% CO_2_ supply. Following adherence, the cell indices were aligned and then treated with different concentrations of cyclosporine A (Enzo Life Sciences, Farmingdale, NY, USA) or tacrolimus (InvivoGen, San Diego, CA, USA) (0.01–10 µM) diluted in EGM-2 with 8% FBS and 1% P/S. Cell indices were continuously monitored for the following 72 h. For all experiments, concentrations of cyclosporine A and tacrolimus were chosen based on those that were described as effective in other publications using in vitro models. These covered anticipated in vivo doses but excluded doses with significant cytotoxicity [42,52,53,94,95].

### 4.3. Chemotaxis Assay

To analyze ECFCs’ ability of directional cell migration in the presence or absence of cyclosporine A or tacrolimus, a modified Boyden chamber assay was performed to determine chemotactic motility. Transwell inserts with an 8 µm microporous membrane (ThinCerts; Greiner, Kremsmünster, Austria) were placed in a 12-well-plate; 5 × 10^4^ ECFCs from 4 cell lines were seeded per transwell insert into the upper side of the chamber in serum-free EBM-2 medium including 1% P/S. FBS (10%) was used as a chemoattractant on the lower side of the chamber. Different concentrations (0.01–10 µM) of cyclosporine A or tacrolimus were added to both sides of the chamber. Dimethyl sulfoxide (DMSO, Sigma-Aldrich, St. Louis, MO, USA) was used as vehicle control. The cells were allowed to migrate for 4 h before the inserts were removed, and the non-migrated cells were detached from the upper surface of the membrane with a cotton bud. The inserts were fixed in 3% *w/v* paraformaldehyde and 2% *w/v* sucrose in phosphate buffered saline (PBS) for 10 min, followed by 2 washing steps with PBS. Afterwards, the migrated cells on the bottom side of the membrane were counterstained with 4′,6-diamidino-2-phenylindole (DAPI, Thermo Fisher Scientific, Waltham, MA, USA), followed by mounting in antifade fluorescence mounting medium (Pro-LongGold; Thermo Fisher Scientific). Five pictures per membrane were randomly taken with a Leica DMI 6000 B microscope (Leica, Wetzlar, Germany). DAPI-stained cells were counted with ImageJ 1.50b (National Institutes of Health).

### 4.4. Migration Assay

A scratch wound healing assay was used to evaluate cell migration; 5 × 10^4^ ECFCs from 4 lines were seeded in duplicates on gelatin-coated (Sigma-Aldrich) wells of 6-well culture plates with EGM-2 containing 10% FBS and 1% P/S and grown to confluence. The cell monolayers were scratched with a sterile P1000 pipette tip to create a wound and washed with PBS. Afterwards, cells were cultured in EBM-2 containing 2.5% FBS and 1% P/S with different concentrations of cyclosporine A or tacrolimus (0.01–10 µM) or vehicle control. Phase-contrast microscopic images were taken immediately after scratching and again after 18 h with a Leica DMI 6000 B microscope. Non-populated scratch areas were quantified by ImageJ 1.50b. The area after 18 h was subtracted from the area at the beginning to obtain the remigrated area.

### 4.5. In Vitro Angiogenesis Assay

To compare the capacity of ECFCs to form capillary tubule-like networks in the presence or absence of cyclosporine A or tacrolimus, 1.4 × 10^4^ cells/well from 4 cell lines were placed in triplicates in 96-well plates pre-coated with 30 µL growth factor reduced Matrigel (BD Biosciences). The cells were incubated for 14 h with different concentrations of cyclosporine A or tacrolimus (0.01–10 µM) or vehicle control in EBM-2 with 1% P/S and VEGF (Thermo Fisher Scientific, 25 ng/mL). The optimal time frame was determined in advance by live cell imaging of untreated ECFCs from healthy subjects and further used in previous studies [96,97,98]. Phase-contrast microscopic images were taken with a Leica DMI 6000 B microscope. Total tube length was calculated with ImageJ 1.50b.

### 4.6. Isolation of Proteins and Immunoblotting

For analyses of proteins, ECFCs from 3–4 cell lines were grown to 80–90% confluence in 10 cm dishes (Sarstedt, Nümbrecht, Germany). For analysis of AKT phosphorylation, ECFCs were incubated with cyclosporine A or tacrolimus (1 or 10 µM) in EBM-2 with 1% P/S (and without FBS or supplements) for 1 h before addition of VEGF (25 ng/mL) for 10 min. For analysis of NF-κB p65 phosphorylation, ECFCs were incubated with cyclosporine A or tacrolimus (1 or 10 µM) for 30 min. TNF-α (10 ng/mL) was used as positive control. ECFCs were then detached with trypsin-EDTA (Bio&Sell), washed with PBS, and lysed with lysis buffer as previously described [93]. Protein concentration was determined by the Bradford protein assay [99]. Proteins were separated by electrophoresis on SDS-polyacrylamide gels and transferred to nitrocellulose membranes (GE Healthcare, Waukesha, WI, USA). After blocking for 1 h with 1% Roti-Block (Carl Roth, Karlsruhe, Germany) in H_2_O, the membrane was incubated overnight at 4 °C with appropriate antibodies (1:1,000 pAKT S473 and 1:1,000 AKT [9271S and 9272S, Cell Signaling, Danvers, MA, USA], 1:1,000 NF-κB p-p65 [sc-136548, Santa Cruz, Dallas, TX, USA], 1:3,000 anti–beta-actin (A54410, Sigma-Aldrich). After washing 3 times with PBS-T (0.1% *v*/*v*), the secondary antibody was added (1:3,000 (or 1:5,000 for beta-actin) goat anti-mouse or goat anti-rabbit horseradish peroxidase; GE Healthcare) in 1% Roti-Block for 2 h at room temperature. Visualization of immunoblot bands was performed by using ECL chemiluminescence (Thermo Fisher Scientific), and analysis was carried out with ImageJ 1.50b.

### 4.7. Quantitative Real-Time PCR (qRT-PCR)

ECFCs from 4–6 lines were treated with cyclosporine A or tacrolimus (1 or 10 µM) for 6 h. In the case of NF-κB inhibition, 10 µM parthenolide (Enzo Life Sciences) was added 1 h prior. RNA was purified using the RNeasy Plus Mini Kit (Qiagen, Hilden, Germany) according to the manufacturer’s instructions. For the cDNA synthesis, RNA was diluted with diethylpyrocarbonate (DEPC)-treated water and denatured at 68 °C for 10 min in a thermocycler (PTC 200, Biozym Scientific GmbH, Hessisch Oldendorf, Germany). Then, High-Capacity cDNA Reverse Transcription (RT) master mix (Applied Biosystems, Waltham, MA, USA) was added. For qRT-PCR, cDNA and master mix (FastStart Universal SYBR Green, Roche) were pipetted into the appropriate strip tubes (0.1 mL). Real-time PCR was performed on a Rotor-Gene 6000 (Qiagen) for 40 cycles. For each treatment, runs were performed in triplicates. The primer sequences used to determine mRNA levels are described in Table 1. Ct values were automatically generated, and relative quantification of gene expression was calculated by standard ΔCt method using the expression of 18S rRNA as reference.

### 4.8. Flow Cytometry

Flow cytometry analysis was performed to measure apoptosis and further to detect adhesion molecule expression on ECFCs’ surface. For apoptosis measurement, ECFCs from 5 lines were harvested with trypsin/EDTA after 48 h of treatment with cyclosporine A or tacrolimus (0.01–10 µM) as described for the cell impedance assay. For adhesion molecule detection, ECFCs from 3–5 lines were treated with 1 µM cyclosporine A or 1 µM tacrolimus for 12 h. For NF-κB inhibition, 10 µM parthenolide (Enzo Life Science) was added 1 h prior to cyclosporine A or tacrolimus treatment. ECFCs were harvested by incubating with accutase (Capricorn, Ebsdorfergrund, Germany) for 15 min at room temperature. In both experimental settings, ECFCs were centrifuged and washed with flow cytometry buffer (PBS, 2% FBS). Then, 1 × 10^5^ cells were blocked with intraglobin (5 mg/mL; Gamunex 10%, Grifols, Frankfurt am Main, Germany) for 1 min, followed by incubation at 4 °C for 30 min with an Annexin V antibody (#640906, Biolegend, San Diego, CA, USA) or with VCAM1 APC (BioLegend), and PECAM1 FITC (BioLegend) antibodies or the corresponding isotype controls, respectively. UV radiation with a transilluminator (Biostep, Jahnsdorf, Germany) for 30 min was used as positive control for apoptosis for gating strategy. For apoptosis analysis, propidiumiodide (PI, Sigma-Aldrich, 10 µg/mL) was added 1 min prior to measurement. Flow cytometry measurements were performed on a BD FACS Calibur Flow Cytometer (BD Biosciences), and results were analyzed using FlowJo X Software v.10 (Tree Star, FlowJo ILC; Ashland, OR, USA).

### 4.9. Immunocytochemistry

Immunocytochemistry was used to demonstrate the expression and localization of NF-κB p65 in ECFCs in the presence or absence of cyclosporine A or tacrolimus. ECFCs from 3 lines were grown to 80–90% confluence on coverslip glasses. After treatment with cyclosporine A or tacrolimus (1 µM) for 30 min, cells were washed with PBS and fixed with 3% *w/v* paraformaldehyde/2% *w/v* sucrose in PBS for 10 min. After washing 3 times with PBS and permeabilization with 0.2% (*v*/*v*) Triton X-100 (Sigma-Aldrich) in PBS, the cells were incubated with an NF-κB p65 antibody (sc-8008 AF488, Santa Cruz, CA, USA) in antibody diluent [2% (*v*/*v*) normal goat serum (Thermo Fisher Scientific), PBS] for 2 h. The cells were washed 3 times with PBS and incubated with Alexa Fluor anti-rabbit IgG 488 (Thermo Fisher Scientific) for 2 h. Nuclear DNA was stained with DAPI, and coverslip glasses were mounted in antifade fluorescence mounting medium. Image acquisition was performed with a Leica DMI 6000 B microscope. For quantification of ECFCs showing nuclear translocation of NF-κB p65, cells were counted in 6 randomly taken images per treatment.

### 4.10. Lactate Dehydrogenase Cytotoxicity Assay

Cytotoxicity was assessed by the release of lactate dehydrogenase (LDH) into the medium. Therefore, the medium of 3 ECFC lines treated with DMSO as vehicle control, cyclosporine A or tacrolimus (1 or 10 µM) for 18 h was collected after the scratch wound healing assay. The LDH measurement was performed as recommended in the manufacturer’s instructions using the in vitro toxicology kit (#TOX7-1KT, Sigma-Aldrich). LDH release was expressed as a percentage of maximum releasable LDH of an equivalent ECFC sample after complete cell lysis obtained by sonification in the same cell culture media used for experimentation (positive control).

### 4.11. Statistical Analysis

Normality distribution was tested by Shapiro–Wilk or D’Agostino normality test and considered before each statistical test. Wilcoxon signed-rank test or one-sample t-test were applied for normalized data, whereas unpaired t-test or Mann–Whitney test were applied for not normalized data. A two-way ANOVA was applied for grouped analyses. Experimental data are presented as median. Additional statistical information, including interquartile ranges (IQR), is placed in the corresponding supplemental tables. The obtained individual measured values (*n*) from each experiment were analyzed with Prism 9 (GraphPad Software, La Jolla, CA, USA). A significant difference is indicated by values of *p* < 0.05, *p* < 0.01, *p* < 0.001.

## Figures and Tables

**Table 1:** Primer sequences for target genes.

Gene	Sense	Antisense
*TNF-α*	CCCAGGCAGTCAGATCATCTT	TCAGCTTGAGGGTTTGCTACA
*IL-6*	GGTACATCCTCGACGGCATCT	GTGCCTCTTTGCTGCTTTCAC
*VCAM*	ATGGTCGTGATCCTTGGAGC	AGATTCTGGGGTGGTCTCGA
*ICAM*	GAACCAGAGCCAGGAGACAC	CTTCACTGTCACCTCGGTCC
*RNA18S1*	ACATCCAAGGAAGGCAGCAG	TTTTCGTCACTACCTCCCCG

## Data Availability

The data that support the findings of this study are available from the corresponding author upon reasonable request.

## Supplementary Materials

The following are available online at https://www.mdpi.com/article/10.3390/ijms22189696/s1.
