# Supplementary material for: Cyclosporine A and Tacrolimus Induce Functional Impairment and Inflammatory Reactions in Endothelial Progenitor Cells
Source: Int J Mol Sci. 2021 Sep 8;22(18):9696. doi: 10.3390/ijms22189696 (PMC8472421; doi:10.3390/ijms22189696)
Supplement: Supplementary file 1 [file ijms-22-09696-s001.zip › Supplemental figures_R1.pdf]

Supplemental Figures

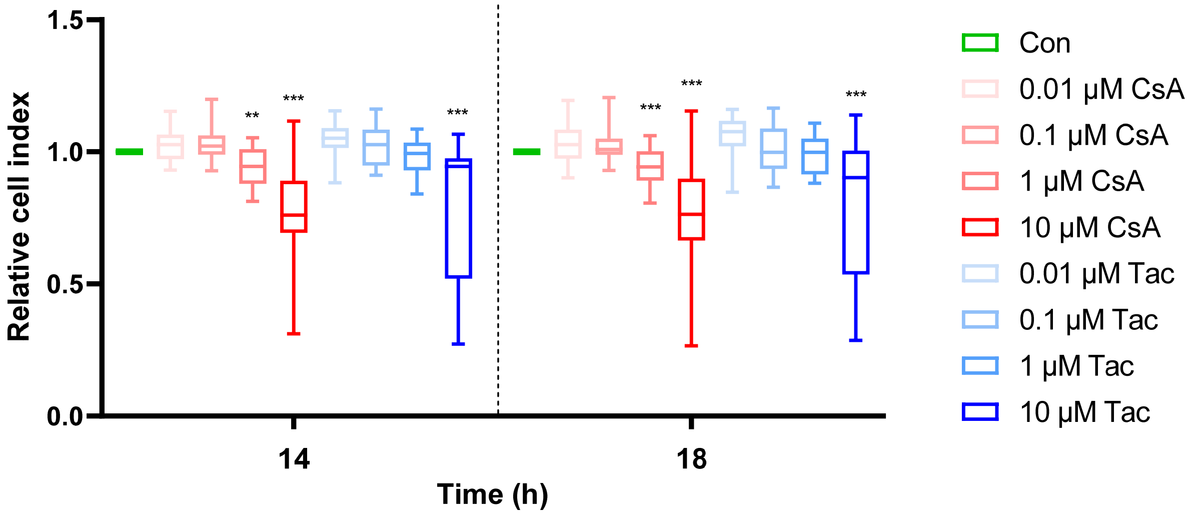

Supplemental Figure S1. High dose cyclosporine A (CsA) and tacrolimus (Tac) impaired ECFC proliferation. ECFC proliferation was significantly decreased after treatment with 1 µM or 10 µM CsA or 10 µM Tac after 14 h and 18 h. n = 13 – 27; control group set as 1. Con, control; CsA, cyclosporine A; Tac, tacrolimus. ** P < 0.01, *** P < 0.001.

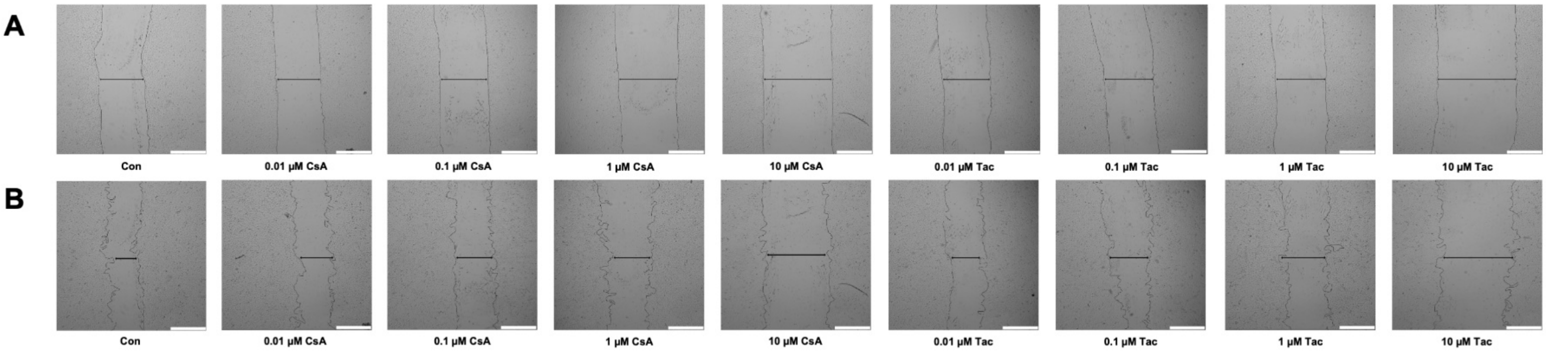

Supplemental Figure S2. Representative images of monolayers with scratch wounds at 0 h (A) and 18 h (B) of incubation with vehicle control only, 0.01 µM CsA, 0.1 µM CsA, 1 µM CsA, 10 µM CsA, 0.01 µM Tac, 0.1 µM Tac, 1 µM Tac, 10 µM Tac, scale bar 1000 µm. Cell-free area after 18 h was subtracted from cell-free area at start to calculate remigrated area.
