# Supplementary material for: Cyclosporine A and Tacrolimus Induce Functional Impairment and Inflammatory Reactions in Endothelial Progenitor Cells
Source: Int J Mol Sci. 2021 Sep 8;22(18):9696. doi: 10.3390/ijms22189696 (PMC8472421; doi:10.3390/ijms22189696)
Supplement: Supplementary file 1 [file ijms-22-09696-s001.zip › Supplemental tables_R1.pdf]

Supplemental Table S1. Statistical analysis of the cell index (24 h).

Treatment	Median	IQR	p	n
0.01 μM CsA	0.99	0.95-1.05	0.84	13
0.1 μM CsA	0.99	0.97-1.04	0.98	15
1 μM CsA	0.94	0.85-0.98	<0.001	26
10 μM CsA	0.75	0.56-0.89	<0.001	26
0.01 μM Tac	1.09	1.00-1.12	0.14	16
0.1 μM Tac	1.05	0.93-1.13	0.68	15
1 μM Tac	0.99	0.89-1.05	0.31	22
10 μM Tac	0.88	0.52-1.00	<0.001	27

Supplemental Table S2. Statistical analysis of the cell index (48 h).

Treatment	Median	IQR	p	n
0.01 μM CsA	1.00	0.96-1.04	>0.99	13
0.1 μM CsA	1.04	0.98-1.08	0.12	15
1 μM CsA	0.93	0.88-0.98	<0.001	26
10 μM CsA	0.73	0.47-0.88	<0.001	26
0.01 μM Tac	1.04	0.97-1.14	0.19	16
0.1 μM Tac	1.03	0.90-1.13	0.80	15
1 μM Tac	0.95	0.89-0.97	0.004	22
10 μM Tac	0.84	0.60-0.94	<0.001	27

Supplemental Table S3. Statistical analysis of the cell index (72 h).

Treatment	Median	IQR	p	n
0.01 μM CsA	1.04	0.97-1.09	0.20	13
0.1 μM CsA	1.06	0.96-1.10	0.14	15
1 μM CsA	0.92	0.87-0.99	0.004	26
10 μM CsA	0.74	0.50-0.87	<0.001	26
0.01 μM Tac	1.08	0.93-1.17	0.18	16
0.1 μM Tac	1.03	0.80-1.13	0.98	15
1 μM Tac	0.90	0.88-0.93	<0.001	22
10 μM Tac	0.84	0.68-0.97	<0.001	27

Supplemental Table S4. Statistical analysis of the cell index (14 h).

Treatment	Median	IQR	p	n
0.01 μM CsA	1.03	0.97-1.07	0.19	13
0.1 μM CsA	1.02	0.99-1.06	0.11	15
1 μM CsA	0.95	0.88-1.01	0.002	26
10 μM CsA	0.76	0.69-0.89	<0.001	26
0.01 μM Tac	1.05	1.02-1.09	0.06	16
0.1 μM Tac	1.03	0.95-1.08	0.39	15
1 μM Tac	0.99	0.93-1.04	0.43	22
10 μM Tac	0.95	0.52-0.98	<0.001	27

Supplemental Table S5. Statistical analysis of the cell index (18 h).

Treatment	Median	IQR	p	n
0.01 µM CsA	1.03	0.98-1.08	0.34	13
0.1 µM CsA	1.01	0.99-1.05	0.21	15
1 µM CsA	0.94	0.89-1.00	<0.001	26
10 µM CsA	0.76	0.66-0.90	<0.001	26
0.01 µM Tac	1.08	1.02-1.12	0.06	16
0.1 µM Tac	1.00	0.94-1.09	0.60	15
1 µM Tac	1.00	0.92-1.05	0.46	22
10 µM Tac	0.90	0.54-1.00	<0.001	27

Supplemental Table S6. Statistical analysis of apoptosis and necrosis – early apoptotic cells (%).

Treatment	Median	IQR	p	n
Con	1.57%	1.51%-1.97%	-/-	5
0.01 µM CsA	2.01%	0.73%-3.21%	>0.99	5
0.1 µM CsA	0.84%	0.64%-2.78%	>0.99	5
1 µM CsA	2.16%	1.28%-4.01%	0.80	5
10 µM CsA	3.40%	2.62%-12.12%	0.57	5
0.01 µM Tac	1.55%	0.81%-18.73%	0.87	5
0.1 µM Tac	1.53%	0.78%-14.18%	0.90	5
1 µM Tac	1.40%	1.05%-14.32%	0.88	5
10 µM Tac	2.60%	1.41%-17.63%	0.83	5

Supplemental Table S7. Statistical analysis of apoptosis and necrosis – late apoptotic cells (%).

Treatment	Median	IQR	p	n
Con	3.26%	2.07%-4.33%	-/-	5
0.01 µM CsA	2.34%	1.84%-4.09%	>0.99	5
0.1 µM CsA	2.10%	1.10%-4.15%	0.98	5
1 µM CsA	3.13%	2.34%-5.14%	>0.99	5
10 µM CsA	5.74%	4.55%-14.67%	0.52	5
0.01 µM Tac	2.68%	1.79%-13.18%	0.96	5
0.1 µM Tac	2.66%	1.40%-9.31%	>0.99	5
1 µM Tac	2.65%	2.38%-10.26%	0.96	5
10 µM Tac	3.94%	2.62%-12.86%	0.87	5

Supplemental Table S8. Statistical analysis of apoptosis and necrosis – necrotic cells (%).

Treatment	Median	IQR	p	n
Con	3.98%	1.98%-4.81	-/-	5
0.01 µM CsA	3.73%	3.09%-5.15%	0.87	5
0.1 µM CsA	4.25%	3.81%-6.11%	0.61	5
1 µM CsA	3.81%	3.04%-5.24%	0.90	5
10 µM CsA	8.50%	5.04%-16.02%	0.31	5
0.01 µM Tac	3.46%	1.71%-4.29%	0.90	5
0.1 µM Tac	3.38%	2.96%-4.69%	>0.99	5
1 µM Tac	3.88%	3.76%-5.66%	0.30	5
10 µM Tac	6.02%	2.51%-6.81%	0.18	5

Supplemental Table S9. Statistical analysis of chemotaxis ability.

Treatment	Median	IQR	p	n
0.01 µM CsA	0.83	0.73-1.01	0.02	20
0.1 µM CsA	0.80	0.59-0.94	<0.001	20
1 µM CsA	0.74	0.69-0.81	<0.001	20
10 µM CsA	0.72	0.57-0.83	<0.001	20
0.01 µM Tac	0.77	0.65-0.99	0.02	20
0.1 µM Tac	0.80	0.62-0.88	<0.001	20
1 µM Tac	0.76	0.65-0.88	<0.001	20
10 µM Tac	0.82	0.67-0.97	<0.001	20

Supplemental Table S10. Statistical analysis of migration ability.

Treatment	Median	IQR	p	n
0.01 µM CsA	0.76	0.64-0.86	<0.001	15
0.1 µM CsA	0.75	0.70-0.86	<0.001	15
1 µM CsA	0.71	0.65-0.95	<0.001	16
10 µM CsA	0.40	0.33-0.68	<0.001	15
0.01 µM Tac	0.81	0.73-0.93	0.003	16
0.1 µM Tac	0.80	0.71-0.95	0.002	15
1 µM Tac	0.77	0.72-0.86	0.002	16
10 µM Tac	0.78	0.65-0.92	<0.001	15

Supplemental Table S11. Statistical analysis of cytotoxicity determined by LDH release (% of positive control).

Treatment	Median	IQR	p	n
Con	25.78%	20.71%-39.46%	-/-	9
1 µM CsA	27.96%	18.20%-33.58%	0.73	9
10 µM CsA	38.43%	18.74%-66.15%	0.61	9
1 µM Tac	28.94%	19.69%-42.73%	0.73	9
10 µM Tac	31.22%	29.20%-41.96%	0.08	9

Supplemental Table S12. Statistical analysis of tube length determined in an *in vitro* angiogenesis assay.

Treatment	Median	IQR	p	n
0.01 µM CsA	0.77	0.66-0.96	0.005	12
0.1 µM CsA	0.67	0.56-0.80	<0.001	12
1 µM CsA	0.57	0.52-0.74	<0.001	12
10 µM CsA	0.47	0.31-0.61	<0.001	12
0.01 µM Tac	0.79	0.72-0.83	0.02	12
0.1 µM Tac	0.72	0.58-0.77	<0.001	12
1 µM Tac	0.68	0.48-0.74	<0.001	12
10 µM Tac	0.47	0.16-0.67	<0.001	12

Supplemental Table S13. Statistical analysis of AKT phosphorylation determined by immunoblot.

Treatment	Median	IQR	p	n
1 µM CsA	0.76	0.71-0.92	0.04	4
10 µM CsA	0.95	0.77-1.01	0.27	4
1 µM Tac	0.70	0.56-0.84	0.03	4
10 µM Tac	0.78	0.69-0.85	0.01	4

Supplemental Table S14. Statistical analysis of TNF-α mRNA expression determined by qRT-PCR.

Treatment	Median	IQR	p	n
1 µM CsA	1.63	1.25-2.11	0.03	5
10 µM CsA	2.94	2.11-4.12	0.01	6
1 µM Tac	1.85	1.25-2.39	0.03	6
10 µM Tac	2.00	1.83-5.54	0.03	6
1 µM CsA + Pa	0.77	0.36-2.35	0.80	4
10 µM CsA + Pa	1.60	0.85-4.88	0.30	4
1 µM Tac + Pa	0.71	0.47-2.24	0.81	4
10 µM Tac + Pa	0.99	0.31-4.44	0.50	4

Supplemental Table S15. Statistical analysis of IL-6 mRNA expression determined by qRT-PCR.

Treatment	Median	IQR	p	n
1 µM CsA	1.67	1.31-3.36	0.03	6
10 µM CsA	1.56	1.20-2.04	0.02	6
1 µM Tac	2.37	1.79-4.64	0.03	6
10 µM Tac	1.65	1.47-2.73	0.03	6
1 µM CsA + Pa	1.01	0.84-1.47	0.58	4
10 µM CsA + Pa	0.87	0.80-4.10	0.46	4
1 µM Tac + Pa	1.63	0.88-2.14	0.21	4
10 µM Tac + Pa	1.02	0.87-1.42	0.56	4

Supplemental Table S16. Statistical analysis of VCAM mRNA expression determined by qRT-PCR.

Treatment	Median	IQR	p	n
1 µM CsA	2.13	1.60-3.17	0.02	6
10 µM CsA	2.09	1.83-2.60	0.01	5
1 µM Tac	2.39	1.80-3.57	0.03	6
10 µM Tac	2.11	1.66-3.66	0.04	5
1 µM CsA + Pa	1.11	0.91-1.22	0.40	4
10 µM CsA + Pa	1.05	0.71-1.20	0.93	4
1 µM Tac + Pa	1.16	0.76-1.65	0.47	4
10 µM Tac + Pa	0.91	0.60-1.09	0.40	4

Supplemental Table S17. Statistical analysis of ICAM mRNA expression determined by qRT-PCR.

Treatment	Median	IQR	p	n
1 μM CsA	1.44	1.25-1.65	0.02	6
10 μM CsA	2.60	1.61-4.03	0.02	6
1 μM Tac	1.90	1.31-2.58	0.01	6
10 μM Tac	2.14	1.69-3.54	0.03	6
1 μM CsA + Pa	1.05	0.83-1.23	0.77	4
10 μM CsA + Pa	1.01	0.79-1.22	0.97	4
1 μM Tac + Pa	1.20	1.02-1.32	0.12	4
10 μM Tac + Pa	0.92	0.79-1.09	0.45	4

Supplemental Table S18. Statistical analysis of PECAM surface expression determined by flow cytometry (subset) (%).

Treatment	Median	IQR	p	n
Con	99.65%	99.00%-99.73%	-/-	6
1 μM CsA	99.80%	99.68%-99.93%	0.04	6
1 μM Tac	99.85%	99.48%-99.93%	0.12	6
Pa	97.80%	94.50%-99.80%	0.30	3
1 μM CsA + Pa	98.00%	97.30%-99.60%	0.30	3
1 μM Tac +Pa	97.20%	96.90%-99.80%	0.27	3

Supplemental Table S19. Statistical analysis of PECAM surface expression determined by flow cytometry (subset shift) (%).

Treatment	Median	IQR	p	n
Con	49.75%	41.98%-53.15%	-/-	6
1 μM CsA	67.20%	48.55%-92.43%	0.047	6
1 μM Tac	68.65%	47.43%-90.95%	0.10	6
Pa	48.60%	22.00%-61.80%	0.69	3
1 μM CsA + Pa	42.50%	24.00%-52.20%	0.33	3
1 μM Tac + Pa	30.60%	22.90%-63.20%	0.46	3

Supplemental Table S20. Statistical analysis of VCAM surface expression determined by flow cytometry (subset) (%).

Treatment	Median	IQR	p	n
Con	70.20%	39.00%-90.75%	-/-	6
1 μM CsA	87.60%	58.65%-95.28%	0.48	6
1 μM Tac	80.10%	46.85%-95.50%	0.75	6
Pa	61.50%	19.70%-83.00%	0.87	3
1 μM CsA + Pa	37.50%	17.50%-73.30%	0.79	3
1 μM Tac + Pa	55.60%	24.70%-83.30%	0.87	3

Supplemental Table S21. Statistical analysis of VCAM surface expression determined by flow cytometry (subset shift) (%).

Treatment	Median	IQR	p	n
Con	45.35%	41.55%-47.40%	-/-	6
1 µM CsA	69.30%	59.63%-80.95%	<0.001	6
1 µM Tac	48.65%	40.45%-65.78%	0.25	6
Pa	50.60%	44.00%-61.00%	0.34	3
1 µM CsA + Pa	36.90%	27.60%-38.80%	0.06	3
1 µM Tac + Pa	55.00%	39.20%-57.80%	0.49	3

Supplemental Table S22. Statistical analysis of NF-κB p65 phosphorylation determined by immunoblot.

Treatment	Median	IQR	p	n
1 µM CsA	1.55	1.17-1.82	0.03	6
10 µM CsA	1.17	1.09-1.37	0.03	6
1 µM Tac	1.39	1.30-2.26	0.03	6
10 µM Tac	1.28	1.16-1.59	0.03	6
TNF-α	1.63	1.39-3.04	0.03	6

Supplemental Table S23. Statistical analysis of NF-κB p65 nuclear translocation (%) determined by immunofluorescence.

Treatment	Median	IQR	p	n
Con	3.13%	0.00%-17.50%	-/-	6
1 µM CsA	88.10%	80.93%-96.59%	0.002	6
1 µM Tac	89.44%	85.61%-92.86%	0.002	6
